# Combined Laser Strategies for Scar Treatment: A Comprehensive Review of Synergistic Protocols

**DOI:** 10.3390/bioengineering12121368

**Published:** 2025-12-16

**Authors:** Alessandro Clementi, Giovanni Cannarozzo, Luca Guarino, Elena Zappia, Fortunato Cassalia, Andrea Danese, Marco Gratteri, Annunziata Dattola, Caterina Longo, Steven Paul Nisticò

**Affiliations:** 1 Department of Dermatology, University of Modena and Reggio Emilia, 41124 Modena, Italy; 2 Department of Clinical Sciences, Sapienza University of Rome, 00185 Rome, Italy; drcannarozzo@gmail.com (G.C.); luca.guarino@uniroma1.it (L.G.); annunziata.dattola@uniroma1.it (A.D.); steven.nistico@uniroma1.it (S.P.N.); 3 Dermatology Unit, Department of Medicine (DIMED), University of Padua, 35121 Padua, Italy; fortunato.cassalia@studenti.unipd.it; 4 Unit of Dermatology, Department of Integrated Medical and General Activity, University of Verona, 37100 Verona, Italy; adanese4@gmail.com; 5 Department of Plastic, Reconstructive and Cosmetic Surgery, Campus Bio-Medico University Hospital, 00185 Rome, Italy; m.gratteri@unicampus.it

**Keywords:** laser therapy, scar management, combined protocols, fractional CO_2_ laser, hypertrophic and burn scars, atrophic scars

## Abstract

Skin scars represent a complex therapeutic challenge, with significant functional, aesthetic, and psychological implications. Despite advances in laser therapy, monotherapy has significant limitations, particularly for patients with complex scars with atrophic, hypertrophic, vascular, and pigmentary components. The combined use of multiple laser sources, in sequential or simultaneous mode, allows for the selective targeting of specific tissue components and improves clinical efficacy while maintaining a good safety profile. This narrative review critically analyses the available evidence on combination therapies for atrophic, hypertrophic, keloid, and post-surgical and burn scars. Protocols combining ablative lasers (CO_2_, Er:YAG), non-ablative lasers (1540–1550 nm), vascular lasers (PDL, Nd:YAG) and intense pulsed light (IPL) are reported. Possible integrations with adjuvant techniques, such as radiofrequency, platelet-rich plasma (PRP), and laser-assisted drug delivery, are also mentioned as areas for future development. The available data suggest a promising role for multimodal strategies, but the literature remains limited by small cohorts, heterogeneous protocols, and short follow-up periods. Although adverse events are generally mild and transient, typically involving erythema, oedema, or temporary dyschromia, an awareness of safety considerations remains essential, particularly in higher phototypes and when using ablative modalities. Further prospective and multicentre studies are needed to define standardised protocols and consolidate the role of combination therapies in the management of scars.

## 1. Introduction

Skin scars are a common result of impaired repair processes following trauma, surgery, burns, or chronic inflammatory diseases. They can compromise not only function and aesthetics, but also have a psychological and social impact, significantly affecting quality of life and interpersonal relationships [1,2].

Atrophic scars result from the loss of dermal tissue and appear as visible depressions on the skin surface [3]. Hypertrophic scars and keloids, on the other hand, are characterised by excessive collagen deposition, with tissue thickening and, in keloids, growth beyond the margins of the wound [4,5]. Burn scars are often extensive, symptomatic, and functionally limiting [6]. Pigmentary and vascular alterations are frequently associated, making the picture even more complex [7].

In recent decades, laser therapy has radically changed the approach to scar treatment. Fractional ablative lasers, such as CO_2_ and Er:YAG, allow for the controlled vaporisation of fibrotic tissue with stimulation of dermal remodelling [8,9]. Fractional non-ablative lasers induce dermal coagulation with faster recovery times [10]. Vascular lasers, such as PDL and Nd:YAG, reduce erythema and hypervascularisation, while intense pulsed light (IPL) offers a versatile approach thanks to its broad emission spectrum [11,12,13].

Preliminary results with emerging wavelengths, such as 675 nm and 1726 nm, suggest potential applications in scar treatment and prevention, and will be subjects of future investigations [14,15].

Safety is a key aspect of all laser procedures. Although combined protocols enhance efficacy, they may increase thermal load and potential side effects such as erythema, oedema, or transient dyschromia. When conservative parameters, proper cooling, and eye protection are used, adverse events remain mild and self-limited. Studies confirm an overall favourable safety profile across ablative, non-ablative, and vascular lasers, particularly under experienced supervision [16].

Despite the progress made, monotherapies still have obvious limitations.

Complex scars combine atrophic, hypertrophic, vascular, and pigmentary elements that are difficult to manage with a single laser source. Each wavelength interacts with a specific chromophore and acts on distinct tissue targets. Vascular lasers such as PDL and Nd:YAG reduce erythema and vascular proliferation through haemoglobin absorption, while ablative lasers like CO_2_ and Er:YAG remodel fibrotic tissue by targeting water and promoting collagen reorganisation. Non-ablative lasers stimulate dermal neocollagenesis, and pigment-specific lasers correct residual chromatic alterations by acting on melanin [12,17].

The sequential or simultaneous use of multiple laser sources allows for the comprehensive treatment of different components within the same session or through temporally coordinated protocols. The underlying hypothesis is that the combination of complementary wavelengths can enhance clinical results by acting simultaneously on different scar components, although the overall safety profile of such combinations requires further evaluation. Furthermore, integration with other technologies, such as radiofrequency, platelet-rich plasma, or exosomes has opened new perspectives for the biological modulation of scar tissue [18,19].

Recent prospective studies have reported encouraging results for combined approaches, including sequential CO_2_ + Nd:YAG, CO_2_ + PDL, and CO_2_ + Er:YAG protocols, with documented improvements in scar texture, colour, and softness compared to monotherapy [16,18]. Despite promising results, the available literature does not yet provide definitive answers regarding the optimal sequence of sources, the duration of induced remodelling, and the stability of results over time, making further investigations with prolonged follow-up necessary.

This review therefore aims to summarise and critically discuss the current evidence supporting such multimodal strategies, outlining their biological rationale, efficacy, and clinical implications.

## 2. Methodology

The bibliographic search was conducted on PubMed and Google Scholar, focusing on randomised trials, prospective studies, and clinical works with at least ten patients, concentrating on combined strategies for various types of scars. The search included articles published up to September 2025 and aimed to provide an updated perspective on combined laser strategies for scar management. Although this narrative review is not a systematic meta-analysis, this structured approach aimed to provide an updated and clinically oriented overview of the current evidence.

## 3. Rationale for Combined Laser and Adjunctive Therapies

The rationale behind combined laser therapies lies in the biological complexity of scar tissue and the intrinsic limitations of monotherapies. Each wavelength interacts with a specific chromophore, according to the principles of selective photothermolysis. This ensures precision and safety but limits the ability of a single source to address the multiple components of the scar [20,21].

Vascular lasers, such as PDL (585–595 nm), selectively target oxyhaemoglobin and allow photocoagulation of the aberrant vessels typical of hypertrophic scars and active keloids [22]. The Nd:YAG 1064 nm penetrates deeper and allows larger calibre vessels to be treated [23,24]. Ablative CO_2_ and Er:YAG lasers act on tissue water, inducing controlled micro-ablations, collagen contraction, and fibroblast stimulation [25,26]. Q-switched and picosecond lasers (532, 755, 1064 nm) fragment melanin pigments, making them useful in treating post-inflammatory discolouration of mature scars [27,28,29]. The main clinical indications for the different wavelengths are summarised in Table 1.

The combined approach allows for the limitations of monotherapy to be overcome. A burn scar, for example, may present with atrophy, hypertrophy, erythema, and discolouration simultaneously [30,31]. No single wavelength can address all these components. Furthermore, the use of aggressive parameters in monotherapy leads to prolonged recovery times and an increased risk of complications such as persistent erythema, infections, or post-inflammatory hyperpigmentation, especially in dark skin types [32,33,34]. A therapeutic plateau is also often observed after a few sessions, with progressively reduced benefits [35].

The combined use of multiple sources, on the other hand, allows for more conservative parameters, maintaining efficacy and reducing adverse events. A classic example is the combination of fractional ablative laser and vascular laser, which allows for the simultaneous treatment of texture and erythema. The microchannels produced by the ablative laser can also facilitate the penetration of other lasers, amplifying its effect [9].

The optimal sequencing of multimodal therapies remains debated. In most studies, vascular lasers such as PDL or Nd:YAG are performed first, followed by fractional CO_2_ laser for textural remodelling. Some authors adopt separate sessions spaced 4–6 weeks apart, while others combine both treatments within the same session, maintaining the vascular–ablative order. In non-erythematous scars, fractional CO_2_ laser is sometimes applied first to induce temporary vascular activation, thereby enhancing the subsequent response to dye laser treatment. However, no consensus exists on ideal timing, highlighting a gap for future research [12,16,36].

Accurate documentation is essential in the treatment of scars. The use of standardised photography, multispectral analysis, and dermoscopy allows for objective and comparable assessments, ensuring clinical reproducibility [36,37,38,39,40]. This approach allows for more precise selection of sources and protocols, optimising therapeutic results. The importance of early intervention should also be emphasised: it is not necessary to wait years before treating scars. On the contrary, the timely use of lasers, particularly dye lasers, has proven effective in the early stages, promoting optimal healing and reducing the risk of progression to hypertrophic or keloid forms [41,42].

Alongside combinations of laser sources, integration with non-laser technologies further expands the possibilities. Radiofrequency induces chromophore-independent volumetric heating, stimulating deep dermal remodelling. PRP, applied after fractional lasers, accelerates healing and reduces recovery times. Laser-assisted drug delivery (LADD) allows corticosteroids or other molecules to be delivered directly into scar tissue [43,44,45,46,47,48]. More recently, exosomes have been proposed as biological adjuvants capable of modulating the scar microenvironment [49]. Preliminary data are promising but need further confirmation in large-scale studies.

## 4. Clinical Evidence for Combined Therapies by Scar Type

Numerous synergistic combinations between different laser sources have been explored, as well as protocols combining lasers and adjuvant therapies. Most of the available evidence comes from studies with small samples and heterogeneous designs, which limit the possibility of drawing definitive conclusions. However, the following sections also report more robust studies that provide more consistent clinical data. The analysis is organised by scar type, and the main results are summarised in Table 2.

### 4.1. Atrophic and Dyschromic Scars

Atrophic scars are among the most common scars treated with lasers. These are noticeable depressions in the skin’s surface, resulting from destructive inflammatory processes such as severe acne, chickenpox, or trauma. In this area, CO_2_ remains the gold standard: controlled ablation, thermal stimulation, and remodelling. Non-ablative wavelengths can add a biostimulating effect, improving the overall response [8].

Kurganskaya and Kluchareva conducted a large prospective study involving 115 patients with atrophic scars. The subjects were divided into two groups based on scar maturity: 49 had lesions in the formation phase and 66 had stabilised scars. In the more recent cases, the protocol involved a sequence of long-pulse Nd:YAG followed by fractional CO_2_, while in mature cases, a combination of Nd:YAG and CO_2_ in planar mode was used. The outcomes were assessed using standardised clinical scales, profilometry, and quality of life indices such as Skindex-29 and DLQI (Dermatology Life Quality Index). The results showed clinical efficacy in 90% of emerging scars and 86% of mature lesions, with tangible improvements in terms of skin elasticity, microcirculation, and micro-relief. Adverse events were limited, mainly transient erythema and pruritus. The authors observed that in recent scars, the main effect is dermoplastic and fibromodulating, while in more stable forms, re-epithelialisation and corrective phenomena prevail. This effect was not evaluated histologically but inferred from objective instrumental parameters: dermal elasticity was quantified through cutometric analysis; microcirculatory changes were measured via laser Doppler flowmetry; and skin profilometry documented progressive normalisation of the micro-relief. The convergence of these findings—improved elasticity, enhanced microcirculation, and smoother surface architecture—was interpreted as indicative of a fibromodulating response. This work represented one of the first structured confirmations of the synergistic potential between Nd:YAG and CO_2_, with an approach modulated according to the stage of the scar [50].

A different contribution comes from the study by Cho and colleagues, who reported clinical experience on 158 patients with facial scars of various origins: acne, chickenpox, trauma, and surgery. The protocol involved a first pass with high-energy CO_2_, followed by Er:YAG 2940 nm for surface finishing. The idea was to exploit the ability of CO_2_ to induce deep collagen contraction and, at the same time, use the ablative precision of Er:YAG to regularise the more superficial layers with less thermal damage. The average follow-up, at approximately ten months, showed a clinical improvement of more than 70% in almost all patients. In 32 cases, the results exceeded 90%, while only five subjects reported improvements of less than 70%. The adverse events reported included persistent erythema in approximately 9% of patients, transient post-inflammatory hyperpigmentation in 14%, and the temporary appearance of hypertrophic scars in just under 20% of cases, all of which resolved without permanent sequelae. The study demonstrated that the sequential combination of CO_2_ + Er:YAG can ensure deep remodelling and surface finishing, balancing efficacy and recovery times [51].

A different approach was described by Ustuner and colleagues in a randomised study involving 49 patients with striae distensae, for a total of 392 lesions evaluated. Each patient underwent two protocols: some striae distensae were treated with fractional CO_2_ monotherapy and others with a sequential combination of CO_2_ and Nd:YAG Q-switched 1064 nm. Three sessions were performed at four-week intervals. The clinical analysis considered parameters such as colour, vascularity, degree of atrophy, width, and length of the lesions. Scales such as GAIS, patient satisfaction indices, and VAS for pain were used. The data showed a more marked reduction in vascularity and atrophy in the combination group, with an average reduction in the width of the striae distensae from 3.6 to 1.6 mm and in length from 48 to 34 mm. The average GAIS score was 1.9 in the combination group versus 1.2 in the monotherapy group, with a statistically significant difference. Patient satisfaction was also higher in the combination group (VAS 7.8 vs. 5.4), albeit with slightly reduced tolerability due to more intense transient pain. The authors concluded that the addition of Nd:YAG allows simultaneous treatment of the atrophic and pigmentary components of striae distensae, producing a more complete clinical improvement [52].

In complex cases, a chromatic component may also appear. In the presence of obvious discolouration, Q-switched or picosecond sources become the instruments of choice. Integration with fractional lasers and vascular sources allows parallel action on pigment and texture [27,28].

Adjuvant techniques such as microneedling, radiofrequency, and LADD further expand the possibilities: delivery of growth factors, biostimulants or depigmenting molecules through ablative microchannels, accelerating regeneration, and reducing recovery times [48,49].

Most studies relied mainly on subjective clinical scales, with limited use of objective instrumental measurements, which represents a methodological limitation when interpreting these results.

### 4.2. Hypertrophic Scars

Hypertrophic scars and keloids represent the opposite end of the scar spectrum. These scars were associated with excess collagen production and raised lesions and were often symptomatic. Hypertrophic scars tend to remain confined to the edges of the wound, with partial regression over time. Keloids, on the other hand, extend beyond the original edges and rarely regress spontaneously.

Treatment depends greatly on the location. In the earlobes, areas of low tension, excision combined with ablative CO_2_ and sometimes dye laser can be effective. The situation is different for areas of high tension (shoulders, sternal region), where the risk of recurrence is high and multimodal protocols with surgery and laser are preferred.

Keshk and colleagues enrolled 30 patients with immature hypertrophic scars in a prospective randomised trial. The protocol included five sessions at monthly intervals with fractional CO_2_, followed in the same session by Nd:YAG long-pulse 1064 nm. Clinical assessment was performed at 3 and 6 months using the Vancouver Scar Scale and Patient and Observer Scar Assessment Scale. Results showed a significant reduction in thickness, erythema, and pruritus. Histological analysis documented more organised collagen fibres, reorganisation of elastic fibres, and reduced epidermal thickness. Adverse events were mild and transient, with no major complications. The authors pointed out that the CO_2_ + Nd:YAG combination is a safe and effective approach in the early stages, acting at different levels of the scar tissue [54].

A different approach was proposed by Zhang and co-workers, who conducted a prospective randomised study of 138 patients with hypertrophic scars. Three sessions at regular intervals compared narrow-band IPL with the combination IPL + fractional CO_2_. One hundred and one patients completed the follow-up. The outcomes, assessed with the POSAS and satisfaction scales, showed a more pronounced improvement in patients treated with the combination, with a reduction in colour, thickness, and stiffness. Satisfaction was complete in the CO_2_ + IPL group, compared to 84% for the cases treated with IPL monotherapy. Adverse events were mild and temporary, including erythema and post-inflammatory hyperpigmentation, with no permanent outcomes. The study confirmed the superiority of the CO_2_ + IPL combination, demonstrating how the integration of ablative lasers and broad-spectrum sources offers more complete results [53].

A further contribution comes from Elrod’s study, which analysed 42 children between 4 and 16 years of age with refractory hypertrophic scars. The protocol involved fractional CO_2_ and 595 nm dye laser in sequence, followed by intralesional corticosteroid infiltration. The sessions, repeated every 6–8 weeks, were clinically evaluated with VSS and POSAS. The results showed an average reduction of 58% and 61%, respectively, with a particular benefit on itching and pain, symptoms often relevant in paediatric age. Tolerability was good: topical anaesthesia was sufficient in most cases, and no serious adverse events occurred. The authors emphasised that the combination of ablative and vascular lasers with drug therapy allows for more complete tissue remodelling and better symptom control, representing a promising strategy even in paediatric patients [55].

The addition of adjuvant techniques remains an important chapter. LADD allows corticosteroids or other molecules to be delivered directly into the scar tissue. Alternatively, lasers can be followed by intralesional injections of botulinum toxin or corticosteroids. Multimodal strategies have been shown to improve outcomes and reduce recurrence in the most refractory forms.

Outcome assessment was predominantly based on subjective scoring systems, and the limited incorporation of objective tools remains a relevant constraint of the available evidence.

### 4.3. Burn Scars

Burn scars are one of the most difficult challenges. Within the same clinical picture, atrophy, hypertrophy, joint contractures, and persistent discolouration can be observed. Significant functional limitations are often added to the aesthetic and psychological damage. Laser therapy can act on both symptoms and functional recovery.

Matuszczak and colleagues conducted a prospective study on 34 adult patients with mature burn scars. The protocol involved five monthly sessions with dye laser followed by fractional CO_2_. Clinical evaluation with VSS and POSAS showed an improvement of more than 50% in over two-thirds of patients. An innovative aspect of the study was the analysis of serological parameters: after treatment, a reduction in collagen I levels and modulation of enzymes involved in extracellular matrix turnover were observed. These data suggest that the effects of laser therapy may extend beyond the treated site, producing systemic modulation. Adverse events were modest, limited to transient erythema and mild pain. The authors concluded that the combination of PDL + CO_2_ is an effective and safe strategy for the treatment of burn scars, with clinical, histological, and biochemical confirmation [56].

Hultman et al. presented one of the largest case series available, including 147 adult patients with extensive burn scars. The protocol involved an initial use of dye laser, followed four weeks later by fractional CO_2_. Cycles were repeated three or four times depending on clinical severity. The results showed a significant improvement in VSS scores, with mean values reduced from 11.3 to 4.8 at six months, maintained up to 24 months. Multivariate analysis identified predictors of success such as age under 40, early initiation of treatment within 18 months of the event, and good adherence to the rehabilitation programme. Tolerability was high, with mild and temporary adverse events. This work confirmed that combined PDL + CO_2_ protocols not only improve aesthetics but also have a positive impact on skin function and quality of life [57].

Another relevant study is that of Zuccaro et al., who described their paediatric experience with 125 children with hypertrophic burn scars, who underwent a total of 289 sessions. Most patients received combined CO_2_ and PDL treatments in the same session, with reduced parameters to ensure safety and tolerability. After a single treatment, the mean VSS decreased from 7.37 to 5.76, with progressive improvements in subsequent treatments. More than half of the children were under 5 years of age at the time of the first intervention, and the results documented not only a reduction in thickness and erythema, but also an improvement in subjective symptoms such as itching and pain. Tolerability was excellent, with topical anaesthesia sufficient in almost all cases and no serious complications. The authors emphasised the importance of early intervention in paediatric patients, taking advantage of the greater regenerative capacity of tissues to prevent functional complications and improve long-term outcomes [58].

Overall, the available evidence confirms that burn scars, in both adults and children, benefit from combined protocols that combine vascular and ablative lasers. The positive impact concerns both morphological and symptomatic and functional aspects, with a favourable safety profile.

Despite favourable findings, most studies used subjective evaluations, and the scarce use of objective measurements continues to limit comparability across protocols.

## 5. Current Limitations and Future Perspectives

Despite documented progress, significant limitations characterise the current literature. The heterogeneity of protocols remains the main challenge, with each centre using empirical parameters based on local experience. This variability includes not only laser parameters (fluence, density, number of passes) but also application sequence, intervals between modalities, and total number of sessions. The consequence is difficulty in comparing results and the impossibility of conducting rigorous meta-analyses.

Limited sample size plagues most studies. Only a few randomised trials with adequate statistical power are available. The prevalence of retrospective series and observational studies limits the level of evidence. Furthermore, most studies come from a single centre, reducing generalisability.

The lack of standardisation in outcome measures further complicates the picture. While VSS and POSAS are frequently used, inter-observer variability and subjectivity limit comparability. Objective measures such as ultrasound, colorimetry, 3D profilometry, or multispectral imaging are rarely employed and should be systematically integrated in future studies to obtain reproducible quantitative data.

The inadequate representation of different populations is a critical gap. Most studies predominantly include Caucasian phototypes I–III. Data on phototypes V–VI are scarce despite the higher prevalence of pathological scars and complications in these populations. Similarly, data on paediatric and geriatric populations are limited.

Insufficient follow-up prevents assessments of long-term efficacy and the identification of late complications. Most studies report a follow-up of 3–6 months, which is inadequate for scars that continue to remodel for 12–24 months. In addition, long-term safety data regarding repeated or sequential laser combinations remain limited and warrant specific evaluation. Some recent studies have explored the adjunctive use of biological agents such as PRP, exosomes, or laser-assisted drug delivery, showing preliminary synergistic effects. However, these remain emerging adjuncts and were only briefly mentioned, as their detailed discussion falls outside the primary focus of this laser-based review.

This review is also limited by its narrative design, which does not adhere to a systematic review methodology. The inclusion of studies was guided by clinical relevance and expert judgement rather than strict predefined criteria, which may have introduced selection bias. Moreover, the substantial heterogeneity in protocols, parameters, and outcome measures prevented a reliable quantitative comparison.

## 6. Conclusions

The evidence available today confirms the value of combined laser strategies in the treatment of pathological scars. This review provides an updated synthesis of the current knowledge on combined laser approaches, emphasising their emerging synergistic role and the rationale for a personalised multimodal strategy in scar management.

The synergy between different wavelengths, combined with the integration of adjuvant techniques, opens therapeutic possibilities that were not achievable with individual methods.

Optimal treatment requires personalisation. It is necessary to start with a thorough understanding of the biology of the scar and the individual characteristics of the patient. A detailed understanding of the different laser sources and their mechanisms of action is a prerequisite for the rational adaptation of therapeutic algorithms. There is no universal protocol: the approach must be modulated according to the type and maturity of the scar, skin phototype, anatomical location, and clinical objectives.

Future research should focus on standardising protocols through multicentre studies, identifying predictive biomarkers of response, and integrating new technologies, including cell therapies and exosomes. Only through systematic and collaborative research will it be possible to fully realise the potential of combined laser therapies and improve the quality of life of patients with pathological scars.

## Figures and Tables

**Table 1:** Main laser sources and clinical indications in scar management.

Laser Type (Wavelength)	Indications in Scar Management
CO_2_ laser (10,600 nm)	Surgical mode: vaporisation of exuberant tissue (hypertrophic scars, keloids). Fractional ablative mode: dermal remodelling, improvement of atrophic scars and hypertrophic and burn scars (texture, pliability, pain). Combination possible with almost all different wavelengths. Adjunctive use: creation of microchannels for laser-assisted drug delivery (corticosteroids, 5-FU, botulinum toxin, PRP, exosomes, etc.).
Er:YAG laser (2940 nm)	Ablative/fractional mode: precise ablation with minimal thermal damage; resurfacing of atrophic facial scars; contour refinement of post-surgical scars. Combination: often used after CO_2_ for surface polishing.
Non-ablative fractional laser (1540–1550 nm)	Dermal heating and neocollagenesis without epidermal ablation; used for mild atrophic scars and striae distensae. Combination: sequential with CO_2_ or other wavelengths for reduced downtime and enhanced remodelling.
Nd:YAG laser (1064 nm)	Long-pulsed mode: treatment of deep vascular component in hypertrophic scars; improvement of erythema, thickness, pliability and biostimulating effect. Combination: adjunct to CO_2_ for multi-layered remodelling.
Fractional Q-switched 1064 nm/Picosecond (1064 nm)	Fractional mode: improvement of scar texture and dermal remodelling through sub-ablative columns; effective also in post-acne scars. Particularly suitable in Fitzpatrick skin types III–V, where ablative lasers carry higher risk of PIH.
Pulsed dye laser (PDL, 585–595 nm)	Vascular targeting: reduction in erythema, vascular hyperplasia, pruritus, and pain in erythematous scars. Combination: often paired with CO_2_ for combined vascular + textural effects.
Intense pulsed light (IPL)	Broad-spectrum vascular/pigment target: reduction in erythema and hyperpigmentation in hypertrophic and post-traumatic scars. Combination: used after fractional lasers for simultaneous modulation of texture and dyschromia.
Q-switched/Picosecond lasers (532, 755 nm)	Pigment fragmentation: correction of post-inflammatory hyperpigmentation and dyschromic scars. Combination: adjunct to fractional CO_2_ for atrophic scars with pigmentary alterations.

**Table 2:** Clinical studies on combined laser therapies for scar management.

Author (Year) and Indication	Combined Lasers	Parameters (as Reported)	Sessions/Interval	Scales and Main Outcomes	Adverse Events
Kurganskaya & Kluchareva (2021)—Atrophic scars [50]	Nd:YAG 1064 nm long-pulse + CO_2_ (fractional/planar)	Nd:YAG long-pulse followed by fractional or planar CO_2_; parameters modulated according to scar stage	NR	Efficacy: 90% (emerging) and 86% (mature); improved elasticity and microcirculation	Transient erythema and oedema
Cho et al. (1999)—Atrophic facial scars [51]	CO_2_ + Er:YAG (sequential)	CO_2_: 250–300 mJ, 50–60 W, 1–3 passes; Er:YAG: 300 μs pulse, 2–5 mm spot; feathering technique	Single combined procedure; follow-up at 3–12 months	Clinical and photographic improvement >70% in majority of patients	Prolonged erythema (9%), PIH (14%), transient hypertrophic scars (18.5%)
Ustuner et al. (2025)—Striae distensae [52]	Fractional CO_2_ + Q-switched Nd:YAG 1064 nm	CO_2_ fractional; Nd:YAG QS 1064 nm; exact fluences not reported	3 sessions, 4-week intervals	Higher GAIS (3.8 vs. 2.9); greater dermoscopic normalisation; higher VAS satisfaction	Mild, transient erythema; no serious complications
Zhang et al. (2023)—Hypertrophic scars (post-trauma) [53]	Fractional CO_2_ + narrow-band IPL	CO_2_ adjusted to scar thickness (30–60 mJ); IPL 560–590 nm, 16–20 J/cm^2^, double pulse 2.4–6.0 ms	5 sessions, monthly	POSAS improvement 45.3% vs. 28.7%; VSS reduction greater in combo; better for <12 months scars	PIH in 8% combo vs. 12% IPL alone
Keshk et al. (2024)—Immature hypertrophic scars [54]	Fractional CO_2_ + Nd:YAG 1064 nm long-pulse	CO_2_ 30–40 mJ/microspot, density 20%; Nd:YAG 40–50 J/cm^2^, 20 ms, 6 mm	4 sessions, every 2 months	VSS reduction 68% vs. 15%; POSAS improved; histology: collagen reorganisation, normalised I/III ratio	Transient erythema and oedema
Elrod et al. (2020)—Paediatric hypertrophic scars [55]	Fractional CO_2_ + PDL 595 nm + intralesional corticosteroids (LADD)	CO_2_ 20–30 mJ/microspot, density 10–15%; PDL 6–7 J/cm^2^, 0.45 ms, 10 mm; triamcinolone 40 mg/mL via LADD	4 sessions, ≥6-week intervals	VSS and POSAS improved; reduction in itch (−72%) and pain (−68%)	Minimal; good tolerability with topical anaesthesia
Matuszczak et al. (2021)—Burn scars (adults) [56]	PDL 595 nm + Fractional CO_2_	PDL 5–10 J/cm^2^; CO_2_ 54–80 mJ/microspot, density 15–25%	4 sessions, 6–8-week intervals	VSS improved (9.2 → 4.0); POSAS improved; biomarkers: Collagen I ↓, MMP-2 ↑, TIMP-1 ↓	Transient erythema; no major complications
Hultman et al. (2013)—Extensive burn scars [57]	PDL 595 nm ± Fractional CO_2_; optional IPL	PDL 5–11 J/cm^2^, 1.5 ms, 7 mm; CO_2_ (UltraPulse): 15 mJ deep @ 600 Hz, 70–90 mJ superficial @ 150 Hz; IPL 515–590 nm, 18–24 J/cm^2^	Multiple sessions, every 4–6 weeks	Improved VSS and UNC4P; sustained long-term outcomes; algorithm described	Transient PIH; caution in darker phototypes
Zuccaro et al. (2018)—Paediatric burn scars [58]	Fractional CO_2_ + PDL 595 nm	PDL 5–9 J/cm^2^; CO_2_ 53–78 mJ core energy (fusion/deep modes)	Variable; 125 patients, 289 procedures	VSS improved 7.37 → 5.76; pigmentation, vascularity, pliability, height improved	Low complication rate; safe in paediatric setting

## Data Availability

The original contributions presented in this study are included in the article. Further inquiries can be directed to the corresponding author.
